# Glial cells: an important switch for the vascular function of the central nervous system

**DOI:** 10.3389/fncel.2023.1166770

**Published:** 2023-05-03

**Authors:** Ling Gao, Xuezhen Pan, John H. Zhang, Ying Xia

**Affiliations:** ^1^Department of Neurosurgery, Affiliated Haikou Hospital, Xiangya School of Medicine, Central South University, Haikou, China; ^2^Department of Physiology and Pharmacology, School of Medicine, Loma Linda University, Loma Linda, CA, United States

**Keywords:** astrocyte, microglia, oligodendrocyte, central nervous system (CNS), blood-brain barrier (BBB), vascular function, neurology

## Abstract

In this review, we first describe the current understanding of glial-mediated vascular function affecting the role of the blood-brain barrier (BBB) in central nervous system (CNS) disorders. BBB, mainly composed of glial and endothelial cells (ECs), is the protective structure that orchestrates the transport of substances, including ions, molecules, and cells from brain vessels into or out of the CNS. Then, we display the multiple communication between glial and vascular function based on angiogenesis, vascular wrapping, and blood perfusion in the brain. Glial can support microvascular ECs to form a blood network connecting to neurons. Astrocytes, microglia, and oligodendrocytes are the common types of glial surrounding the brain vessel. Glial-vessel interaction is required for the permeability and integrity of BBB. Glial cells surrounding the cerebral blood vessels can transmit communication signals to ECs and regulate the activity of vascular endothelial growth factor (VEGF) or Wnt-dependent endothelial angiogenesis mechanism. In addition, these glial cells monitor the blood flow in the brain via Ca^2+^/K^+^-dependent pathways. Finally, we provide a potential research direction for the glial-vessel axis in CNS disorders. Microglial activation can trigger astrocyte activation, which suggests that microglia-astrocyte interaction may play a key role in monitoring cerebral blood flow. Thus, microglia-astrocyte interaction can be the key point of follow-up studies focusing on the microglia-blood mechanism. More investigations focus on the mechanism of how oligodendrocyte progenitor cells communicate and interact with ECs. The direct role of oligodendrocytes in modulating vascular function needs to be explored in the future.

## Introduction

Central nervous system (CNS) disorder is a broad group of diseases characterized by a lack of intact nervous system circuits. Blood-brain barrier (BBB) dysfunction is the core pathological basis promoting the occurrence and development of CNS disorders. BBB plays a neuroprotective role in the brain structure. BBB controls the transport of physical and chemical signals into and out of the CNS (Sweeney et al., 2019), which protects the brain from disruption by injurious substances including neurotoxic blood-derived debris, cells, and microbial pathogens (Sweeney et al., 2018). Glial are the main components of BBB, which have contact with the brain vessels and control the extracellular and intracellular signals to communicate with vascular cells such as endothelial cells (ECs) (Alvarez et al., 2013). Neurons, glial, ECs, and pericytes form a three-dimensional functional unit named the neurovascular unit (Zhao et al., 2021). Therefore, the homeostasis of the CNS is affected by the significant relationship between glial and brain vessels.

Scientific interest in the glial-mediated pathogenesis targeting brain vessel dysfunction in CNS disorders is rapidly progressing. In the present review, we first described the current understanding of glial-mediated vascular function affecting the role of BBB in CNS disorders. We showed the significance of BBB dysfunction in CNS disorders. Then, we displayed the multiple communication between glial and vascular function based on angiogenesis, vascular wrapping, and blood perfusion in the brain. We introduced the various types of glial cells and discuss the role of glial-ECs interaction. Finally, we provided a potential research direction for the glial-vessel axis in CNS disorders.

## BBB: a natural barrier of the CNS

The central nervous system (CNS) consists of the brain and spinal cord, in which biological functions receive sensory information, process information, and output responding signals (Thau et al., 2022). A dysfunction in the brain or spinal cord will result in CNS disorders such as hypoperfusion, autoimmune dysfunction, brain tumor, traumatic injury, brain metabolic disorder and other unknown injuries.

Owing to the significance and vulnerability of the CNS, the body can form some barrier mechanisms to protect the brain. BBB is the protective structure that orchestrates the transport of substances, including ions, molecules, and cells from brain vessels into or out of the CNS (Profaci et al., 2020). Glial (i.e., astrocytes, microglia, and oligodendrocytes) and blood capillaries consisting of ECs are the common components of blood-brain barrier (BBB) at the cellular level (Sun et al., 2021). Endothelial cells (ECs) in BBB tightly arrange to form the capillary to control material transportation such as iron, insulin, and blood sugar (Chiou et al., 2019; Yazdani et al., 2019). There are influx and efflux. various glial surrounding the brain capillary. Glial can mediate the communication between capillaries and neurons. Thus, BBB serves as the crucial maintainer of CNS homeostasis because of its function in substance.

## Glial-ECs interaction: the support of BBB

In blood-brain barrier (BBB), glial support microvascular ECs to form a blood network connecting to neurons. Glial activation is the vital mechanism of BBB disruption. Astrocytes, microglia, and oligodendrocytes are the common types of glial surrounding the brain vessel, which make contact with ECs through their foot-like structures that perform the glial-related regulation in brain vessels. The neurovascular unit (NVU) is the key functional unit of BBB that consists of ECs, pericytes, glial (astrocytes, microglia, and oligodendrocytes), and neurons. In the structure of the NVU, pericytes wrap around ECs that form the blood vessel wall (Langen et al., 2019); astrocytes are located between ECs and neurons and use their end-feet to communicate with ECs and neurons, respectively; microglia are capable of interacting with ECs based on purinergic pathways, and also extend their “feet” to astrocytes (Liu et al., 2020; Csaszar et al., 2022); intriguingly, oligodendrocyte progenitor cells (OPCs) can migrate to the specific region along the brain blood vessels, whereas oligodendrocytes attach to the synapse of neurons via their end-feet (Nelson et al., 2016). The neurovascular unit (NVU) is the core structure in which glial show significant interaction with ECs in BBB. These glial-ECs interactions transmit neuronal signals to brain vessels to modulate the cerebral blood circulatory system. Correspondingly, the interruption and impairment of glial-ECs interaction contribute to BBB dysfunction.

## Astrocyte-EC interaction

Astrocyte is the most abundant type of glial in the central nervous system (CNS) that are characterized by a star shape and exist in both invertebrates and vertebrates. Based on the structure and location of astrocytes, protoplasmic and fibrous astrocytes are classified as two major cell types. Protoplasmic astrocytes are found in gray matter (Zhou et al., 2019). However, fibrous astrocytes occur in white matter (Kohler et al., 2021). Astrocytes in different regions, even within the same site, show heterogeneity in morphological characteristics and biological functions such as metabolism pathways and gap junction (de Majo et al., 2020; Kohler et al., 2021). Astrocytes play a significant role in maintaining ECs interaction. A study by Shan et al. (2019) concluded that the expression of tight junction proteins (TJP) in microvascular ECs was negatively related to vascular endothelial growth factor (VEGF) A matrix metalloproteinase-9 (MMP-9), chemokine monocyte chemoattractant protein-1 (MCP-1), and chemokine C-X-C motif ligand 1 (CXCL-1) expressed in astrocytes stimulated by oxygen-glucose deprivation (OGD). The activation of astrocytes induces pro-inflammation factors like IL-1β, IL-6, and TNF-α upregulation and decreases GDNF secretion, and then reduces the TJP of ECs (Huang et al., 2022). Astrocytes modulated Delta-like Notch ligands 1 and 4 (Dll1 and Dll4) expression in ECs *in vitro*; Dll4 decreased ECs excessive sprouting and protected ECs integrity (Martinez-Lozada and Robinson, 2020). The intriguing relationship suggests impaired astrocytes influence ECs interaction by affecting the TJP expression in ECs, which promotes BBB dysfunction. Besides the role of astrocytes in EC-EC interaction, astrocytes can also directly block their interaction with ECs. In BBB, the end-feet of astrocytes enwrap the periphery of the vascular structure (Mills et al., 2022). Based on a three-dimensional microvascular model of BBB with ECs, astrocytes are found to attach to the brain vessel consisting of microvascular ECs via the end-feet (Campisi et al., 2018). Astrocytes around the vessel interact with ECs via special communication forms such as calcium signals and extracellular vesicles (EVs) (Braet et al., 2001; Upadhya et al., 2020). The astrocyte-EC interaction is also modulated by the shuttle mechanism of glutamyl-cysteinyl-glycine (GSH). A study based on the metabolic profile in astrocytes showed GSH shuttle from astrocytes to ECs, and the secretion of GSH was increased under OGD stimulation in astrocytes. GSH suppressed tyrosine phosphorylation, which induced ROS accumulation and led to redox homeostasis in ECs and then induced the aberrant phosphorylation of TJP in BBB (Huang et al., 2020). Possibly, the Wnt pathway contributes to astrocyte-EC interaction. The team of Guerit et al. (2021) found that the suppression of the Wnt gene in astrocyte increases caveolin-1 expression in ECs and change the localization of water channel Aqp4 and astrocytic end-feet which is related to the loss of end-feet structural integrity and decreased pericyte. Thereby, the Astrocytic Wnt pathway is responsible for protecting astrocyte-EC interaction and BBB integrity (Guerit et al., 2021). Astrocyte ablation interferes with tight junction function, and the consequent BBB dysfunction is hardly repairable; therefore, astrocytes are necessary for BBB integrity, of which a loss cannot be sufficiently compensated by other cell types (Heithoff et al., 2021).

## Microglia-EC interaction

Microglia are macrophages located in the brain as a part of the mononuclear phagocyte system (Prinz et al., 2019). Microglia specifically express C-X3-C Motif Chemokine Receptor 1 (CX3CR1), CD11b, ionized calcium-binding adaptor molecule 1 (IBA-1), and F4/80, and account for 5–12% of cells in the CNS (Hickman et al., 2018). CX3CR1 can modulate the expression of a subset of inflammatory genes to regulate the phenotype change between activated and resting microglia (Gyoneva et al., 2019). CD11b function involves cell adhesion and cellular uptake for complement-coated particles. IBA-1 located in the cytoskeleton and cell membrane contributes to membrane ruffling and phagocytosis in activated microglia. F4/80 is a glycoprotein on the surface of resting microglia that modulate cell-cell adhesion. The four factors have been considered as biomarkers of microglia. In the central nervous system (CNS), microglia perform the functions associated with immune monitoring, cellular fragment phagocytosis, synaptic plasticity, oligodendrocyte differentiation, and astrocyte activation/proliferation (Wright-Jin and Gutmann, 2019). The perivascular localization of microglia contributes to a significant biofunction of microglia that the cells identify and clean the blood-borne substances and potential inflammatory stimuli (Liebner et al., 2018). In the normal condition, resting microglia (M0 phenotype) function as the immune monitor in the brain; however, microglia can be activated to two polarization phenotypes, classically activated microglia (M1 phenotype promoting inflammation) and alternatively activated microglia (M2 phenotype inhibiting inflammation) in the pathological condition (Zeng et al., 2018). Intriguingly, microglia polarization affects the permeability and integrity of BBB (Yang et al., 2022). Pro-inflammatory microglia contribute to the high permeability of BBB, whereas anti-inflammatory cells protect BBB via the production of IL-10 and TGF-β1 (Ronaldson and Davis, 2020). More concretely, there is a biological communication between microglia and ECs to modulate BBB homeostasis. For instance, recent evidence showed vascular cell adhesion molecule 1 upregulation in brain ECs-activated microglia, thereby promoting neuronal injury and cognitive deficits in aged rats (Yousef et al., 2019). Also, an investigation speculating about the mechanism of BBB disruption in ischemia stroke reported that M1 microglia contribute to the necroptosis in ECs via the tumor necrosis factor (TNF)-α dependent pathway (Chen et al., 2019). Lipopolysaccharide-stimulated microglia have been found to inhibit endothelial function via CXCL13/C-X-C motif chemokine receptor (CXCR) 5 and p38 pathways (Zhang et al., 2022). A report by Haruwaka et al. (2019) showed another role of microglia in BBB integrity. First, they established that the expression of claudin-5 in microglia-mediated microglia-ECs interaction, thereby contributing to BBB integrity. Then, they found microglia could swallow astrocytic end-feet to disrupt BBB permeability. This study indicates microglia can affect BBB function by both microglial and microglial-astrocytic interactions with ECs and suggests that these interactions may not be independent of each other. The EC-associated communication of microglia suggests its distinctive role in CNS diseases via vessel-related mechanism.

## Oligodendrocytes-EC interaction

Under the control of intracellular and extracellular signals, oligodendrocyte progenitor cells (OPCs) migrate to a pre-determined place through vessels in the CNS, then proliferate and differentiate into oligodendrocytes of 6–8 μm in diameter (Karasek et al., 2004; Xia and Fancy, 2021). Oligodendrocytes are able to modulate myelin generation and axonal degeneration (Kuhn et al., 2019). Besides the myelin-forming function, oligodendrocytes also provide nutritional support to the nervous system through lactate release.

Oligodendrocyte dysfunction contributes to the occurrence and development of neurological disorders. For example, Pandey et al. (2022) showed oligodendrocytes developed disease-associated 1 (related to inflammation genes), disease-associated 2 (contributes to genes involved in survival), and disease-associated 3 (influencing the interferon response genes) activation states in the mouse model of Alzheimer’s disease (AD) and multiple sclerosis. The three states affect the oligodendrocyte-involved remyelination in diseases. Intriguingly, recent studies show the potential relationship between Parkinson’s disease and oligodendrocytes. Agarwal et al. (2020) reported Parkinson’s disease risk was associated with oligodendrocyte-specific genes expressed in the cortex and substantia nigra based on single-nuclei sequencing. Smajic et al. (2022) found that oligodendrocytes were decreased in idiopathic Parkinson’s disease. Particularly, they observed that S100B was significantly increased in the remaining oligodendrocytes in response to stress. These reports suggest an oligodendrocyte-related etiology of Parkinson’s disease.

Blood-brain barrier can be interrupted by oligodendroglial dysfunction. In the BBB structure, oligodendroglial-EC interaction is necessary for the maintenance of BBB integrity. OPCs can migrate toward the brain vessel to modulate the permeability of ECs and the tight junction between astrocytes and ECs (Niu et al., 2019; Kimura et al., 2020). In the white matter, the elevation or the reduction in OPCs cause significant changes in vessel density; meanwhile, OPCs make contact with ECs via signaling pathways such as the Wnt-dependent pathway (Chavali et al., 2020). OPCs have a great number of protrusions that play a key role in oligodendroglial-vascular interaction. In the brain of C57BL/6J young mice, a protrusion of OPCs was found to be in contact with multiple vessels, and similar structures were observed in capillaries and arteries (Pfeiffer et al., 2021). Importantly, OPCs failure to detach from brain vessels can also disturb astrocyte-ECs interaction to induce BBB dysfunction under the pathological condition of multiple sclerosis (Niu et al., 2019). Regrettably, the evidence based on the direction of oligodendrocyte-ECs is limited. More investigations focus on the mechanism of how OPCs communicate and interact with ECs. It may be related to research methods and the distribution and function of oligodendrocytes.

Glial cells serve as the core part of the BBB structure to protect the CNS. Glial-vessel interaction is required for the permeability and integrity of BBB. These glial cells (e.g., astrocytes, microglia, and oligodendrocytes) provide the morphological support for vascular function to enhance the communication between vessels and brain parenchyma. Glial-ECs interaction provides an answer to explain how glial cells modulate vascular function during BBB dysfunction.

## Glial and vascular function

As mentioned above, there are some types of Glial-ECs interaction or communication in BBB. These significant relationships finally affect vascular function in the brain, which causes changes in the structure and function of BBB. We explore how the three glial cells (astrocytes, microglia, and oligodendrocytes) modulate cerebral vascular function based on angiogenesis and blood perfusion.

## Glial and angiogenesis

Angiogenesis is a complex process where new blood vessels grow from the existing vasculature, which is necessary for the selective uptake of nutrients and metabolites in all tissues (From the American Association of Neurological Surgeons [AANS], American Society of Neuroradiology [ASNR], Cardiovascular and Interventional Radiology Society of Europe [CIRSE], Canadian Interventional Radiology Association [CIRA], Congress of Neurological Surgeons [CNS], European Society of Minimally Invasive Neurological Therapy [ESMINT], et al., 2018). When the brain takes in and processes the information, it generates a mass of metabolic requirements. Metabolic activity alters the vascular proportion, which is a cause of angiogenesis in the brain (Adair and Montani, 2010). In CNS disorders, the changes in angiogenesis affect cerebral metabolism and nervous survival. As a result of physiological angiogenesis, the brain vessels are grouped as the basic structure of BBB formation (Vallon et al., 2014). Thus, altered angiogenesis contributes to BBB disruption.

The formation and development of the cerebral blood system are accompanied by the differentiation and maturation of glial cells, suggesting the potential regulatory mechanism of glial in the blood vessel. There is an increasing interest in the role of glial in angiogenesis. The role of glial in angiogenesis is heterogeneous. The proliferation and migration in ECs are partly regulated by glial cells.

The increase of hypoxia-inducible factor (HIF) has been determined to induce angiogenesis. The study by Yuen et al. (2014) showed that HIF 1/2α expressed in OPCs could stimulate the proliferation in ECs via the paracrine effect, which ultimately causes angiogenesis in the white matter. They also showed that OPCs produce HIF1/2α to control their own maturation via the Wnt/β-catenin pathway. Based on the ICR mouse model of transient middle cerebral artery occlusion, Wang et al. (2022) concluded that OPCs transplantation can produce Wnt7a to enhance β-catenin expression in ECs, thereby contributing to the capability of functional angiogenesis. It provides evidence that OPCs can modulate angiogenesis in ECs via the Wnt/β-catenin pathway. However, the role of OPCs in angiogenesis is mediated by VEGF rather than the Wnt pathway according to a recent report by Zhang et al. (2020). The two opposite findings may not contradict each other. There may be two pathways, Wnt-dependent and Wnt-independent, for OPCs to regulate white matter angiogenesis. Nevertheless, the direct relationship between oligodendrocytes and angiogenesis still needs further exploration. Oligodendrocytes possibly inherit some of the angiogenesis mechanism from their precursors.

Interestingly, Zhang et al. (2020) also found that astrocytes could activate the Wnt/β-catenin pathway target genes *Axin2* and *Notum* in ECs by astrocytes HIFα to evoke angiogenesis in the brain. The Wnt-related mechanism between astrocytes and ECs is determined in the Nhe1 Astro-KO mice with ischemic stroke (Song et al., 2021). They observed Nhe1 deficiency in astrocytes elevated Wnt 7a/7b and activated the Wnt/β-catenin pathway in ECs, thereby inhibiting BBB permeability and vascular leakage. They established that Nhe1 deficiency mediated the changes in astrocytes from “response” to “protective.” A novel report showed that the loss of Fat1 cadherin disrupted the migrative polarity and maturation in the astrocyte progenitor, which failed to maintain the formation and development of new blood vessels. They think the loss of Fat1 cadherin delayed astrocyte progression by the suppression of platelet-derived growth factor (PDGF)α and VEGF expression, which finally impacts vascular angiogenesis (Helmbacher, 2022).

One function of microglia is to promote angiogenesis both in normal and pathological conditions. A study focusing on the biological behaviors of ECs co-cultured with lipopolysaccharides (LPS)-stimulated microglia showed the potential role of microglia in angiogenesis (Ding et al., 2018). This study found that LPS-stimulated microglia enhanced VEGF-A and PDGF-BB in ECs, which promoted proliferation and migration in ECs. Moreover, TGF-β1-induced M2 phenotype can mediate the microglial regulation in endothelial angiogenesis. An investigation constructing the *in vitro* endothelial model via co-culture with cortical neurons undergoing OGD stimulation showed TGF-β1 enriched in microglia-derived extracellular vesicles promoted M2 polarization in microglia to build the anti-inflammatory microenvironment for ECs, and meanwhile it could also activate Smad2/3 pathway in ECs to induce angiogenesis in the brain (Zhang et al., 2021). This finding not only suggests the therapeutic role of microglia-derived EVs but also demonstrates the microglia-related response to cerebral ischemia.

**FIGURE 1:**
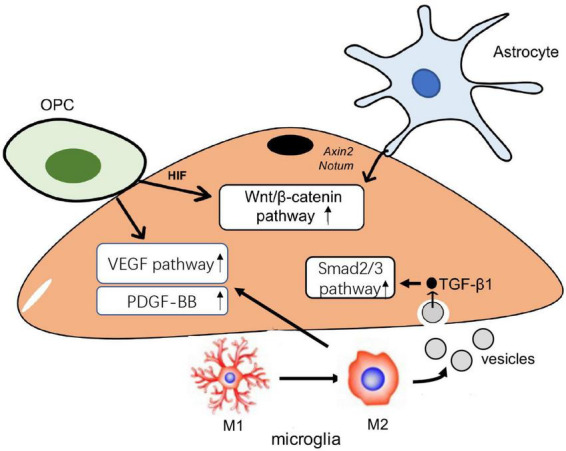
The role of glial in angiogenesis. Oligodendrocyte progenitor cells (OPCs) can activate Wnt/β-catenin and vascular endothelial growth factor (VEGF) pathway to cause functional angiogenesis. Protective astrocytes elevate endothelial Wnt/β-catenin pathway to promote angiogenesis in the brain. Microglia transmit TGF-β1 enriched in extracellular vesicles (EVs) into endothelial cells (ECs) and then activate Smad 2/3 pathway to induce the proliferation and migration in ECs; microglia also modulate the VEGF pathway to drive the angiogenesis.

Collectively, the expression of HIFα in OPC regulates angiogenesis via VEGF; meanwhile, Astroglial HIFα mediates angiogenesis through the Wnt/β-catenin pathway, and microglia also modulate VEGF during M1 to M2 polarization. This evidence indicates that VEGF and the Wnt pathway are important factors of angiogenesis regulated by the glial. VEGF is a critical factor in angiogenesis and regulates vascular permeability and the migration of ECs. The Wnt pathway is involved in angiogenesis, as established in many studies (Vallée et al., 2018), but microglia also participate in angiogenesis via the Smad2/3 pathway and PDGF-BB secretion. The role of glial cells in angiogenesis mainly involves three pathways in ECs, including Wnt/β-catenin, VEGF, and Smad2/3 pathways (Figure 1). By regulating the activation of these pathways, glial cells can modulate the process of angiogenesis to affect the vascular function in the CNS.

## Glial and blood flow

Ischemia cerebral injury is one of the major causes of central nervous system (CNS) diseases, indicating that blood perfusion plays a significant role in CNS homeostasis (Feske, 2021). Mounting reports find that hypoxia can alter proliferation and activation in glial cells [e.g., astrocytes (Perelli et al., 2021) and microglia (Butturini et al., 2019)], suggesting there is a possible mechanism between glial and blood perfusion.

Astrocytes wrap cerebral blood vessels through the end-foot in which various electron transport chain proteins are expressed (Stokum et al., 2021). The astrocytic end-foot provides the physiological basis to involve ion-related regulation in blood flow. Cortical astrocytes regulate blood flow and vasodilation in response to neuronal activity via a Ca^2+^-dependent pathway (Takano et al., 2006). The experimental evidence demonstrated astrocytes produced and sent out prostaglandins and epoxyeicosatrienoic acids into the brain vessels to induce vasodilation via a Ca^2+^-dependent pathway in response to adenosine signaling during hypoglycemia (Nippert et al., 2022). Intriguingly, the Ca^2+^ activity of astrocytes can also promote cerebral vasoconstriction in addition to vasodilation. Boily et al. (2021) used angiotensin II (Ang II) to stimulate astrocytes. They found Ang II could elevate Ca^2+^ in astrocytes end-foot to enhance vasoconstriction via the AT1 receptor, the receptor of Ang II. The K^+^-dependent pathway is another mechanism of astrocyte-blood coupling (Newman et al., 1984; Paulson and Newman, 1987; MacVicar and Newman, 2015). Given the role of astrocytes in blood flow, there is a view that astrocytes can serve as the receptor of cerebral perfusion and activate autonomic sympathetic control circuits in the CNS to provide enough flow in the brain via a compensatory mechanism associated with the increase of heart rate and arterial blood pressure (Marina et al., 2020).

The role of microglia in blood flow has remained unclear for a long time, but it is now determined to be the “switch” in cerebral capillaries. In a C57BL/6J mouse model undergoing chronic mild hypoxia, microglia depletion significantly caused the vascular leak in the white/gray matter, suggesting the potential role of microglia in cerebral blood flow (Halder and Milner, 2019). The experimental evidence by Bisht et al. (2021) first demonstrated that the depletion of capillary-associated microglia, a microglial type (one in three of total microglia) enriched in brain capillaries, could increase vascular diameter and blood flow in the capillaries, which was partly related to purinergic receptor P2Y and G protein coupled 12 (P2RY12)/pannexin1 (PANX1) pathway. In the disease-induced perfusion disorder (e.g., hypercapnia-induced vasodilation), calcium homeostasis in microglia was interfered with and then the impaired microglia failed to respond to cerebrovascular adaptation because of P2RY12 dysfunction, which resulted in hypoperfusion (Csaszar et al., 2022). However, the further mechanism in microglia-blood flow needs to be explored. For example, the observation that microglia can trigger astrocyte activation indicates a hypothesis that the activation of microglial due to the pathological condition could activate astrocytes to increase or decrease cerebral blood flow (Jha et al., 2019; Zong et al., 2020). Also, microglia dysfunction possibly involves the disruption of astrocyte-vessel coupling.

Oligodendrocytes are sensitive to blood flow. A report by Riddle et al. (2006) targeting the relationship between periventricular white matter injury (PWMI) and cerebral ischemia concluded that ischemia condition is caused by PWMI in different grades, which is not simply attributed to the heterogeneity of the degree of ischemia. They further found that oligodendrocyte susceptibility to ischemia condition was the cause of the uneven distribution of PWMI. In other words, oligodendrocyte degeneration is positively associated with the susceptibility to ischemia, and the heterogeneous distribution of advanced oligodendrocytes results in the various susceptibility to ischemia in different regions. We may even speculate that oligodendrocytes can function as ischemia biomarkers in the detection of cerebral ischemia disease using imaging technology. A study investigating oligodendrocyte heterogeneity in the young and the aged brain provided a novel mechanism that expounded the modulation of ischemia condition in oligodendrocytes (Dai et al., 2020). This study demonstrated that the STAT3-mediated protection of TGF-α could inhibit ischemia-induced death in oligodendrocytes. However, the abovementioned studies only discuss the susceptibility of oligodendrocytes to blood flow. Whether oligodendrocytes regulate the blood flow of the brain is still unclear. Oligodendrocyte cell lines include OPCs and oligodendrocytes are extremely sensitive to environmental conditions. For example, OPCs alter their proliferation, migration, and differentiation in response to oxygen tension (Janowska et al., 2018). The oxygen sensitivity of OPCs readily finds the changes in blood flow. There may be a high-sensitive oxygen tension receptor on the membrane of OPCs. Oligodendrocytes may develop the responsive phenotype to reverse the changes in blood flow. More evidence is needed to determine whether sensitive oligodendrocyte cell lines are required for the remodeling of blood flow. Also, the exact mechanism of how oligodendrocytes perceive the changes in blood flow remains under-investigated.

**FIGURE 2:**
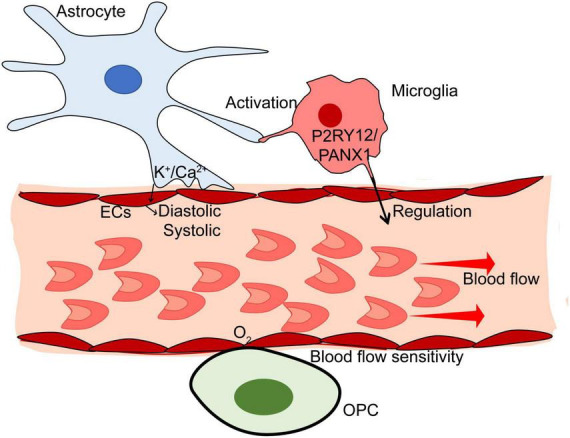
The mechanism of glial in blood flow of the brain. Astrocytes monitor the blood flow based on Ca^2+^/K^+^-dependent pathways. Microglia can regulate the blood flow via P2RY12/PANX1 pathway. Also, activated microglia may modulate cerebral blood flow through astrocyte-related pathways under pathological conditions. Oligodendrocytes are sensitive to blood flow. There may be a high-sensitive oxygen tension receptor on the membrane of Oligodendrocyte progenitor cells (OPCs).

Studies focusing on the glial-mediated regulation mechanism of blood flow are rapidly developing. The role of glial cells in blood flow is heterogeneous (Figure 2). The relationship between glial and blood perfusion provides a novel insight into glial-mediated vascular function in CNS disorders. First, the glial would be the biomarker to monitor the changes in cerebral blood flow based on some of the imaging methods. The regions with abnormal perfusion could be observed by labeling the molecular biomarker of glial cells. Then, targeting impaired glial will be instrumental in the treatment of altered blood flow in the pathological condition, which finally contributes to the therapy for CNS disorders.

## The role of glial-mediated BBB dysfunction in CNS diseases

Blood-brain barrier dysfunction is associated with the occurrence and development of central nervous system (CNS) disorders. BBB dysfunction also causes microenvironment disorder in the CNS (Bosche et al., 2020). In the C57BL/6 mouse model infected with *Toxoplasma gondii*, proinflammatory cytokines including interferon α (IFN) and TNF in the serum infiltrated into the brain tissue because of BBB dysfunction with permeability disruption and triggered glial-mediated neuroinflammation to evoke the changes in the CNS (Castano Barrios et al., 2021). A clinical study focusing on BBB dysfunction in immunotherapy with CD19-specific chimeric antigen receptor (CD19 CAR) T cells suggests the treatment may result in permeable BBB that fails to inhibit the influx of systemic cytokines into the cerebrospinal fluid (Gust et al., 2017). An endothelial transcriptome profile based on RNA-sequencing showed that BBB dysfunction occurred in the *in vivo* and *in vitro* models of stroke, multiple sclerosis, traumatic brain injury, and seizure, with profound changes in gene expression in ECs (Munji et al., 2019). As the major component of BBB, glial impairment is involved in the neurodegeneration progression in CNS disorders. Astrocytes, microglia, and oligodendrocytes modulate the barrier function of BBB during the onset and progression of CNS disorders. Altered glial induce BBB dysfunction with high permeability, which permits harmful substances to invade the CNS.

## BBB dysfunction in Alzheimer’s disease

Alzheimer’s disease is a common memory impairment pathologically characterized by neuritic plaques and neurofibrillary tangles. There are two main hypotheses in the pathogenesis of AD including cholinergic and amyloid hypotheses. The two hypotheses seem to be based on BBB dysfunction. Increasing studies have found that anticholinergic agent influx binds to muscarinic [M(1)] receptors in the CNS because of BBB dysfunction, which results in cognitive deficits (e.g., Alzheimer’s disease) (Chancellor et al., 2012). The phenomenon provides a novel insight into the BBB-mediated cholinergic deficit during Alzheimer’s disease. In the early stage of AD, BBB disruption promotes the extravasation of fibrinogen to enter the brain, thereby leading to oxidative stress and glial-mediated neuroinflammation in the CNS, which may contribute to the formation of amyloid plaque (Leng and Edison, 2021; McLarnon, 2021). Improving BBB dysfunction may be instrumental in inhibiting cholinergic disorder and amyloid accumulation that is induced by high permeability. Some cholinergic modulators (e.g., Vitamin B12) have been found to ameliorate BBB dysfunction (El-Mezayen et al., 2022). BBB dysfunction in Alzheimer’s disease involves glial differentiation. For example, Habib et al. (2020) identified abundant disease-related astrocytes in aging mice with Alzheimer’s disease-like symptoms. They concluded that amyloid plaques activated disease-associated astrocytes in the progression of AD. The specific phenotype of astrocytes may impair NVU-related communication in BBB, which contributes to AD pathogenesis.

## BBB dysfunction in Parkinson’s disease

Blood-brain barrier dysfunction is related to the occurrence and progression of Parkinson’s disease. Patients with Parkinson’s disease characterized by tremor, rigidity, bradykinesia/akinesia, and postural instability lack dopaminergic neurons with the occurrence of Lewy bodies and the depigmentation of substantia nigra pars compacta (Balestrino and Schapira, 2020). Systemic inflammation is involved in the onset and progression of Parkinson’s disease (Pajares et al., 2020). At the pathological level, inflammation in the disease may be attributed to BBB dysfunction. There is a serious leakage of BBB in Parkinson’s disease (Al-Bachari et al., 2020). Based on the meta-analysis, a systemic review concluded that BBB dysfunction combined with other neurodegenerative disorders induces Parkinson’s disease (Wong et al., 2022). A clinical study observed the outside of serum proteins and erythrocytes in the brain parenchyma of patients with Parkinson’s disease (Gray and Woulfe, 2015). The brain with natural barrier loss due to BBB dysfunction fails to resist the exogenous injury, which promotes neuroinflammation. Astrocyte is essential for BBB integrity in Parkinson’s disease. Lan et al. (2022) observed that the increased vascular endothelial growth factor-A (VEGF-A) in astrocytes resulted in BBB damage with decreased expressions of tight junction-related genes and increased permeability. Microglia also take part in the inflammation around BBB in Parkinson’s disease. The interaction between microglia and dead neurons causes oxidative stress, mitochondrial dysfunction, neuroinflammation, and a-synuclein accumulation, which drives BBB dysfunction and Parkinson-like symptoms (Badanjak et al., 2021).

## BBB dysfunction in traumatic brain injury

Blood-brain barrier dysfunction can contribute to blood content extravasation and subsequent inflammation. The main mechanism of brain injury is neurodegeneration, which is caused by oxidative stress in BBB followed by neuroinflammation (Moretti et al., 2015). BBB dysfunction has been observed in the brain of patients with traumatic brain injury. O’Keeffe et al. (2020) observed that BBB dysfunction occurred both in acute and chronic stages of traumatic brain injury, and it was related to mild, moderate, and severe brain injury. They also concluded that the severity of BBB dysfunction depends on the exposure level of head trauma. Moreover, mice undergoing closed-head injury develop BBB dysfunction with altered cerebrovascular reactivity (Ichkova et al., 2020). Recent studies show that the activation of astrocytes and microglial after traumatic brain injury causes dysregulation of IBA1, GFAP, MMP-9, AQP4, and ZO-1, which aggravate BBB dysfunction (Dunn et al., 2021; George et al., 2022). Gao et al. (2022) showed that PD-L1 expression in reactive astrocytes regulates the cavity size of the brain and inflammatory infiltration in the CNS via the CCL2 pathway.

## BBB dysfunction in cerebral ischemia

In stroke, the lack of tight junction proteins (TJP) in ECs evokes blood-brain barrier (BBB) dysfunction with hyperpermeability around the peri-infarct areas, which enlarges the neuroinflammation and neuronal damage (Sulhan et al., 2020; Yang and Torbey, 2020; Candelario-Jalil et al., 2022). BBB dysfunction in stroke is characterized by microglia polarization and dysregulated expressions of tight junction-related genes and transport proteins (Abdullahi et al., 2018; Qiu et al., 2021). The changes in tight junction-related genes cause the increased permeability of BBB, and dysregulated transport proteins affect the substances in the cerebral microenvironment, thereby promoting microglia polarization and modulating neuroinflammation in the brain.

## BBB dysfunction in intracerebral hemorrhage

Intracerebral hemorrhage results in pathological alterations in blood-brain barrier (BBB). Vessel breakdown impairs the structure of BBB and then blood contents enter the CNS via impaired BBB, which contributes to brain injuries such as cerebral edema and inflammatory response. BBB dysfunction in intracerebral hemorrhage involves glial in the brain. Astrocytic conversion plays a role in the post-inflammatory response of intracerebral hemorrhage (Fei et al., 2022). Astrocyte-related post-inflammation further aggravates BBB leakage and enhances permeability. Microglia are related to BBB dysfunction in intracerebral hemorrhage. It has been observed that intracerebral hemorrhage mice with microglia replacement treatment showed improvement in BBB dysfunction (Li et al., 2022).

## BBB dysfunction in brain tumor

Both primary and metastatic brain tumors are accompanied by blood-brain barrier (BBB) dysfunction. The impaired BBB in brain tumors is called the blood-tumor barrier (BTB) with a higher permeability than normal BBB (Mo et al., 2021). In the progression of brain tumors, glial can promote angiogenesis via vascular remodeling and disrupt BBB function. Huang et al. (2014) showed that the upregulation of platelet-derived growth factor receptor α (PDGFRα) led to the activation and recruitment of OPCs that blocked the astrocytic inhibition to ECs, thereby contributing to the ECs-related neovascularization in glioma. Also, microglia can polarize to the pro-inflammatory phenotype when the focal opening of BBB occurs in glioblastoma (Zhang et al., 2023), suggesting the interaction between microglia and BBB in brain tumors. Research on glioblastoma reveals that microglia release IL-6 in the microenvironment of glioblastoma multiforme, disrupt BBB integrity by activating Janus kinase/signal transducer and activator of transcription 3 in ECs, and downregulate TJP (Couto et al., 2019).

## BBB dysfunction in Huntington’s disease

There is a significant relationship between oligodendrocytes and Huntington’s disease. Lim et al. (2022) found oligodendrocytes and oligodendrocyte progenitor cells (OPCs) in Huntington’s disease stopped in the medium mature state. The stagnation in oligodendrocytes and OPCs is capable of enhancing MMP-9 expression that contributes to BBB leakage and inflammatory infiltration (Seo et al., 2013). Vignone et al. (2022) established that microvascular ECs isolated from Huntington’s disease showed abnormality in endothelial barrier function based on the brain microvascular endothelial cell model. Also, brain vessels in Huntington’s disease were characterized by altered vascular density and increased BBB leakage according to a clinical investigation (Denis et al., 2019). BBB dysfunction results in a physiological defect and breaks the balance in the environment around the neurons, which functions as the cause of CNS diseases.

## Effect of permeable BBB in the treatment of CNS diseases

Central nervous system diseases can alter the physical and chemical properties of BBB. In the progression of CNS disorders, an increase in interleukin 1β (IL-1β), TNF-α, and other proinflammatory cytokines causes intracellular factors such as p38 mitogen-activated protein kinase (MAPK), nuclear factor κ B subunit (NF-κB), and Rho-associated coiled-coil containing protein kinase (ROCK) in brain microvascular ECs to induce the increased permeability of ECs (Takata et al., 2021; Waller et al., 2022). The increase of ECs-related permeability result in BBB dysfunction with hyperpermeability. Moreover, BBB limits drug delivery because of the selective permeability although it can prevent toxic substances from getting into the brain in normal conditions. Recent advances in drug delivery focus on modified brain-penetrating peptides and BBB shuttle peptides. A complex drug-loading type conjecting brain penetrating peptide or BBB shuttle peptide with drug molecules provides a novel insight into CNS diseases targeting BBB. For instance, the antiviral porphyrins are covalently attached to the BBB shuttle peptide, which forms a low-cytotoxic complex to overcome the limited permeability of BBB (Mendonca et al., 2021).

Collectively, BBB is a specific frontier linking circulating blood with brain parenchyma, in which permeability disruption and other vascular dysfunction allow hazardous substances to enter the brain and participate in the progression of CNS disorders. Glial can support the barrier function of BBB. Impaired glial play a key role in BBB-related CNS disorders. Improving glial-mediated BBB dysfunction could contribute to the recovery of patients with CNS diseases and then the normal transportation of nutrients and metabolites are rebuilt, which is instrumental in repressing the progression of CNS diseases.

## Conclusion

In general, there are various communication modes between glial cells and cerebral vessels, which is the basis for glial cells to regulate cerebral vascular function and mediate central nervous system (CNS) diseases. It is important to understand that glial-vessel interaction contributes to excavating the novel therapeutic target of BBB dysfunction during CNS diseases. On the one hand, glial cells surrounding cerebral blood vessels can transmit communication signals to ECs and regulate the activity of the VEGF- or Wnt-dependent endothelial angiogenesis mechanism. On the other hand, these glial cells monitor the blood flow in the brain via Ca^2+^/K^+^-dependent pathways. Microglial activation due to the pathological condition may activate astrocytes to increase or decrease cerebral blood flow. Thus, microglial-astrocyte interaction can be the key point of follow-up studies focusing on the microglia-blood mechanism. Besides, the direct role of oligodendrocytes in modulating vascular function needs to be explored in the future.

## Author contributions

LG and XP drafted the manuscript and drew the figures. YX and JZ worked on the manuscript revision and editing. All authors contributed to the conceptualization and approved the final manuscript.
